# Transient interruption of contrast on CT pulmonary angiography: effect of mid-inspiratory vs. end-inspiratory respiration command

**DOI:** 10.1007/s10140-024-02227-z

**Published:** 2024-04-18

**Authors:** Koichiro Yasaka, Masumi Mizuki Takahashi, Mariko Kurokawa, Takatoshi Kubo, Yusuke Watanabe, Hiroyuki Saigusa, Osamu Abe

**Affiliations:** Department of Radiology, Graduate School of Medicine, The University of Tokyo, 7-3-1 Hongo, Bunkyo-ku, Tokyo, 113-8655 Japan

**Keywords:** CT pulmonary angiography, Transient interruption of contrast, Pulmonary artery, CT attenuation, Multivariable analysis

## Abstract

**Purpose:**

To investigate the effects of mid-inspiratory respiration commands and other factors on transient interruption of contrast (TIC) incidence on CT pulmonary angiography.

**Methods:**

In this retrospective study, 824 patients (mean age, 66.1 ± 15.3 years; 342 males) who had undergone CT pulmonary angiography between January 2021 and February 2023 were included. Among them, 545 and 279 patients were scanned at end- and mid-inspiratory levels, respectively. By placing a circular region of interest, CT attenuation of the main pulmonary artery (CT_MPA_) was recorded. Associations between several factors, including patient age, body weight, sex, respiratory command vs. TIC and severe TIC incidence (defined as CT_MPA_ < 200 and 150 HU, respectively), were assessed using logistic regression analyses with stepwise regression selection based on Akaike’s information criterion.

**Results:**

Mid-inspiratory respiration command, in addition to patient age and lighter body weight, had negative association with the incidence of TIC. Only patient age, lighter body weight, female sex, and larger cardiothoracic ratio were negatively associated with severe TIC incidence. Mid-inspiratory respiration commands helped reduce TIC incidence among patients aged < 65 years (*p* = 0.039) and those with body weight ≥ 75 kg (*p* = 0.005) who were at high TIC risk.

**Conclusion:**

Changing the respiratory command from end- to mid-inspiratory levels, as well as patient age and body weight, was significantly associated with TIC incidence.

## Introduction

Pulmonary embolism is the third most common cause of death from cardiovascular disease, following heart attack and stroke [1], and 300,000 people in the United States reportedly die of acute pulmonary embolism [2]. Multidetector CT pulmonary angiography is an established modality for the evaluation of pulmonary embolism with a sensitivity and specificity of 83–100% and 89–97%, respectively [3]. However, the quality of CT pulmonary angiography is degraded in some patients by a phenomenon known as transient interruption of contrast (TIC) [4]. In TIC, while a high concentration of contrast material can be observed in both the superior vena cava and the aorta, contrast enhancement in the pulmonary artery is reduced. This phenomenon is caused by the increased flow of blood from the inferior vena cava during inspiration maneuvers during CT scan [5].

Researchers have endeavored to improve the quality of CT pulmonary angiography in patients with TIC. Recent studies focused on the use of low-keV monochromatic images [6] and low tube voltages [7]. However, obtaining low-keV monochromatic images requires a dual-energy CT scanner, which is less accessible than a conventional single-energy CT. A low-tube-voltage image is associated with increased noise [8]. Furthermore, these approaches only mitigate the problems associated with TIC and do not reduce their incidence. There exist studies which reported that TIC incidence can be reduced by changing the respiratory commands during CT pulmonary angiography. However, results regarding the effects of changing respiratory commands have been contradictory. Some researchers have reported that respiratory coaching before deep inspiration is not effective in preventing TIC [9, 10], while others have reported that eliminating deep inspiration before image acquisition is effective in preventing TIC [11]. One reason for these contradictory results may be the relatively small number of patients included in their studies. Furthermore, while TIC incidence is inversely correlated with patient age [10], no study has investigated the combination of significant factors associated with TIC using multivariable analysis and the effect of changing respiratory commands in high-risk patient groups.

Herein, we evaluated the relationship between various factors including changes in respiratory commands and TIC, and assessed the impact of changes in respiratory commands on TIC incidence in high-risk patient groups.

## Materials and methods

This retrospective study was approved by our institutional review board, which waived the requirement for written informed consent.

### Patients

**Fig. 1:**
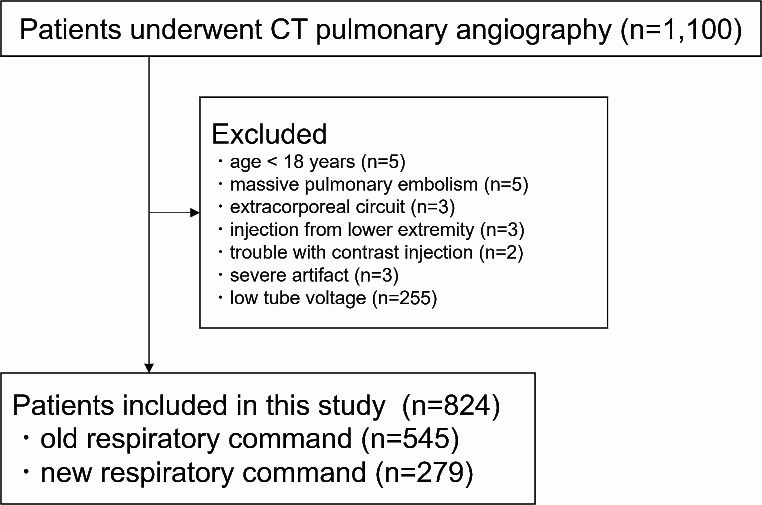
Flowchart of the patient inclusion and exclusion processes

We searched a picture archiving and communication system (Advantage Reporting Version 5.2.10.133 [J-MAC SYSTEM, Hokkaido, Japan]) for patients who had undergone CT between January 2021 and February 2023 by using the term of CT pulmonary angiography, pulmonary artery, and pulmonary embolism. Excluded patients were those : (a) aged < 18 years (*n* = 5), (b) with massive pulmonary embolism hindered measuring the CT attenuation of the pulmonary artery (*n* = 5), (c) on extracorporeal circuit (*n* = 3), (d) injected with contrast materials from the lower extremity (*n* = 3), (e) with trouble in a contrast injection maneuver (*n* = 2), (f) with severe artifacts (*n* = 3), and (g) who underwent CT examination with tube voltage of 100 kVp (single-energy CT pulmonary angiography was started to be scanned with 100 kVp from mid-2022) (*n* = 255). The inclusion and exclusion processes are illustrated in Fig. 1.

### CT imaging

The patients underwent CT examinations using CT scanners from two vendors (Aqulion ONE and Aquilion Prime from Canon Medical Systems, Tochigi, Japan and Discovery CT 750 HD and Revolution CT from GE Medical Systems, WI, US). The tube voltage and current were as follows: fast kV switching dual-energy CT with 70 keV monochromatic image, which corresponds to a 120 kVp image [12], with noise index set at 11.4 (GE-CT [Revolution CT]); 120 kVp with noise index set at 11.36 (GE-CT [Discovery 750 HD]); and 120 kVp with automatic tube current modulation standard deviation set at 13.0 (Canon-CT [Aquilion ONE and Aquilion Prime]). The scan direction was cranio-caudal. CT images were reconstructed at a slice thickness/interval of 5 mm/5 mm for both Canon-CT and GE-CT. For clinical purpose, thinner slice thickness images (3 mm for Canon-CT and 2.5 mm for GE-CT) and thicker slice thickness images (5 mm for both Canon-CT and GE-CT) were reconstructed. However, to unify the slice thickness, we have chosen latter images for the main analyses.

The contrast material (600 mgI/kg) was injected from the right or left antecubital vein within 30 s using an automatic power injector. The flow rate, which was around 3 ml/s, was variable based on patient’s body weight. The intravenous catheter with gauge of 22G was used. The iodine density of contrast material (Iomeprol 350 mgI/mL [Bracco], iopamidol 370 mgI/mL [Bayer], Iohexol 350 mgI/mL [GE Healthcare], or Ioversol 320 mgI/mL [Guerbet]), which was determined based on patient’s body weight (320, 350, and 370 mgI/mL for those with < 48 kg, 49–63 kg, and > 64 kg, respectively in principle), was recorded. The scan begun 20 s after the start of contrast material injection.

At our institution, the respiratory command for CT pulmonary angiography was changed in January 2022. Before January 2022, the patients were scanned at the end-inspiratory level (old respiratory command). After January 2022, patients were given respiratory instructions before CT examination to maintain mid-inspiration levels during CT pulmonary angiography (new respiratory command).

### Quantitative image analysis

A radiologist (radiologist A) with 13 years of imaging experience placed circular regions of interest with diameters of approximately 10 mm on the main pulmonary artery and descending aorta. Subsequently, CT attenuation of the main pulmonary artery (CT_MPA_) and descending aorta (CT_AORTA_) was recorded. Because CT attenuation within the superior vena cava was highly heterogeneous, we did not measure them. To evaluate the reproducibility of the measurement, another radiologist (B, with imaging experience of 6 years) performed those measurement in randomly selected 25 patients. Following a previous report [13], patients with CT_MPA_ < 200 Hounsfield unit (HU) and 150 HU were considered to have TIC and severe TIC, respectively. A radiologist measured the cardiothoracic ratio (CTR) on the scout images.

### Qualitative image analysis

Three radiologists (readers 1/2/3 with diagnostic imaging of 12/10/6 years, respectively) were involved in qualitative image analysis. Before this evaluation, radiologist A randomized all images. The three readers were blinded to the patient background and CT scanning information. They evaluated the quality of images using a four-point scale (1 = severe TIC and nondiagnostic, 2 = TIC degraded image quality although a diagnosis can eventually be made, 3 = TIC is slightly present but no significant reduction in the quality of the study, 4 = TIC is not visually apparent). To evaluate the interobserver agreement, scoring was also performed by another radiologist in randomly selected 75 patients. Therefore, 899 (= 824 + 75) patient data in total were evaluated by 3 radiologists (299–300 patient data by each radiologist).

### Statistical analyses

Statistical analyses were performed using R version 4.1.2 (https://r-project.org/). Nominal and continuous variables were compared using Fisher’s exact test and Student’s *t*-test (or one-way analysis of variance for multiple groups), respectively. Intraclass coefficient for CT_MPA_ and CT_AORTA_ was calculated. For ease, iodine density of contrast agent was dichotomized to ≤ 350 mgI/mL and > 350 mgI/mL in the analyses. *P*-values < 0.05 were considered to indicate statistically significant differences. Associations between age and CTR vs. CT_MPA_ were assessed using Pearson’s correlation coefficients. To determine the combination of parameters associated with CT_MPA_ and TIC incidence, multiple regression analysis and logistic regression analysis, respectively were performed. In these analyses, factors with *p*-values < 0.05 in univariable analyses were used as input data, and stepwise regression selection based on Akaike’s information criterion was performed. The qualitative image score was compared between old and new respiratory command with the Mann-Whitney U test. The interobserver agreement in the qualitative image analysis was calculated with the Cohen’s weighted kappa analysis.

## Results

### Background information

Detailed patient information is shown in Table 1. In this study, 824 patients (mean age, 66.1 ± 15.3 years; 342 males) were included. Among them, 545 (mean age, 65.5 ± 15.6 years; 221 males) and 279 (mean age, 67.4 ± 14.5 years; 121 males) patients underwent CT pulmonary angiography with old and new respiratory commands, respectively. There was no statistically significant difference in patient backgrounds (*p* > 0.080). TIC was observed in 48 (39 and 9 in old and new respiratory protocols, respectively) and severe TIC was observed in 11 (9 and 2 in old and new respiratory protocols, respectively). The CT attenuation of the main pulmonary artery and aorta in old and new respiratory commands were shown in Table 2. Intraclass coefficient (with 95% confidence interval) for CT_MPA_ and CT_AORTA_ was 0.996 (0.990–0.998) and 0.966 (0.992–0.998), respectively.

**Table 1:** Patient baseline information

	All	Old	New	*p*-value
Number of patients	824	545	279	N/A
Age (years)	66.1 ± 15.3	65.5 ± 15.6	67.4 ± 14.5	0.080
Body weight (kg)	59.2 ± 14.6	59.7 ± 13.9	58.5 ± 16.0	0.276
CTR (%)	49.6 ± 6.5	49.9 ± 6.6	49.1 ± 6.5	0.114
Sex (Male / Female)	342/482	221/324	121/158	0.455
Side of injection (Left / Right)	228/596	151/394	77/202	1.000
Contrast agent (≤ 350 / >350 mgI/ml)	590/234	387/158	203/76	0.625
Pulmonary embolism (Present / Absent)	164/660	112/433	52/227	0.580

Note. Number of patients provided is for nominal variables. Means and standard deviations were described for continuous variables. Comparisons between the old and new respiratory commands for nominal and continuous variables were performed using Fisher’s exact test and Student’s *t*-test, respectively. CTR, cardiothoracic ratio; N/A, not applicable

**Table 2:** CT attenuation of the main pulmonary artery and aorta in old and new respiratory commands

	Old respiratory command	New respiratory command
CT_MPA_ (HU)	340.6 ± 110.4	360.2 ± 104.4
CT_AORTA_ (HU)	284.6 ± 80.6	286.2 ± 82.9

### Effect of each factor on CT_MPA_ using univariable analysis

**Fig. 2:**
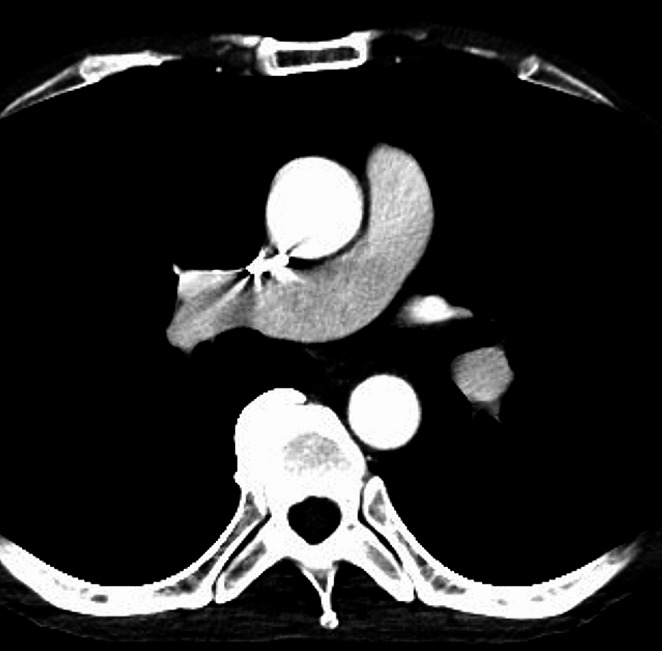
CT pulmonary angiography of a 48-year-old male in whom transient interruption of contrast was seen. The contrast material was injected from right upper extremity in this patient. The CT attenuation of pulmonary artery and descending aorta was 144 and 277 Hounsfield unit, respectively. While high-attenuation contrast material can be seen in both the superior vena cava and aorta, CT attenuation of pulmonary artery is relatively low. This phenomenon would be possibly due to mixture of blood from left upper extremity and inferior vena cava, whose CT attenuation is low, with the inspiration maneuver during scan

**Fig. 3:**
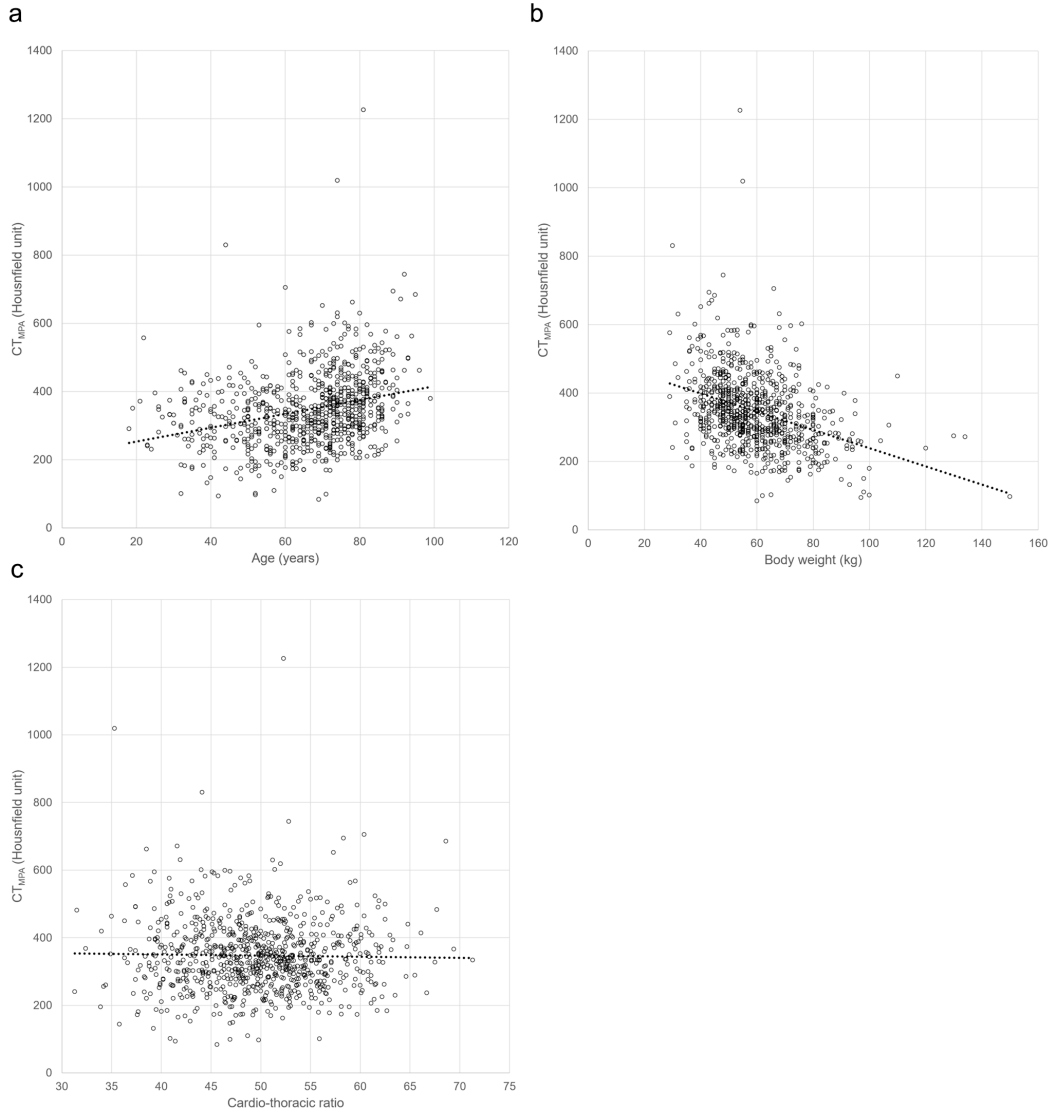
Association between CT_MPA_vs. (**a**) age, (**b**) body weight, and (**c**) cardio-thoracic ratio. Regression lines are described with dotted line. CT_MPA_ = CT attenuation of the main pulmonary artery

Representative CT pulmonary angiography image of a patient with TIC is shown in Fig. 2. The detailed results for the effect of each factor (other than CT scanner) on the CT_MPA_ are provided in Table 3. There was no statistically significant difference in the CT_MPA_ among scanners (342.9 ± 110.5 HU [Canon-CT], 365.1 ± 109.8 HU [Discovery 750 HD], and 354.7 ± 102.5 [Revolution CT], *p* = 0.187). There was a statistically significant positive correlation between age and CT_MPA_ (*r* = 0.284, *p* < 0.001) (Fig. 3a). There was a statistically significant negative correlation between body weight and CT_MPA_ (*r* = -0.358, *p* < 0.001) (Fig. 3b). There was no significant correlation between CTR and CT_MPA_ (*r* = -0.020, *p* = 0.568) (Fig. 3c). Statistically significant difference in CT_MPA_ was also observed for sex (337.0 and 354.5 HU for male and female, respectively [*p* = 0.023]) and iodine density of contrast agent (362.5 and 308.9 HU for ≤ 350 and > 350 mgI/mL, respectively [*p* < 0.001]). The new respiratory command (360.2 HU) significantly increased the CT_MPA_ compared with the old command (340.6 HU, *p* = 0.015).

**Table 3:** Effect of each factor (other than CT scanner) on CT_MPA_

	Values		*p*-value
Correlation coefficient (95% CI) †			
Age	0.284 (0.220–0.346)		< 0.001*
Body weight	-0.358 (-0.416 – -0.297)		< 0.001*
CTR	-0.020 (-0.088 – -0.048)		0.568
CT_MPA_ (Hounsfield unit)			
Sex (Male / Female)	337.0 ± 117.8	354.5 ± 101.2	0.023*
Respiratory command (Old / New)	340.6 ± 110.4	360.2 ± 104.4	0.015*
Side of injection (Left / Right)	349.6 ± 124.3	346.3 ± 102.2	0.697
Contrast agent (≤ 350 / >350 mgI/mL)	362.5 ± 109.9	308.9 ± 95.7	< 0.001*

Note. – Unless otherwise specified, means ± standards deviation are described for continuous variables. Comparisons were performed using the Fisher’s exact test and Student’s *t*-test for nominal and continuous variables, respectively. CT_MPA_ = CT attenuation of the main pulmonary artery; CTR = cardiothoracic ratio

† A Pearson’s correlation coefficient analysis was performed

* Statistically significant

### Effect of each factor on TIC incidence using univariable analysis

Detailed results of the effect of each factor (other than CT scanner) on TIC incidence are provided in Table 4. There was no statistically significant difference in the incidence of TIC among scanners (6.6% [Canon-CT], 1.8% [Discovery 750 HD], and 4.6% [Revolution CT], *p* = 0.291). There was a statistically significant difference in age between patients with TIC (54.7 years) and those without (66.8 years, *p* < 0.001). There was also a statistically significant difference in body weight between patients with TIC (74.0 kg) and those without (58.4 kg, *p* < 0.001). The TIC incidence in male patients (8.5%) was significantly higher than that in female patient (3.9%, *p* = 0.010). The TIC incidence in patients who received > 350 mgI/mL (10.7%) contrast agent was also significantly higher than ≤ 350 mgI/mL (3.9%, *p* < 0.001). The new respiratory commands helped reduce the incidence of TIC (3.2%) compared to that associated with the old command (7.2%, *p* = 0.027).

**Table 4:** Effect of each factor (other than CT scanner) on TIC incidence

	Values		*p*-value
TIC (CT_MPA_ < 200 HU)			
Age (years) (TIC+ / TIC–)	54.7 ± 11.6	66.8 ± 15.2	< 0.001*
Body weight (kg) (TIC+ / TIC–)	74.0 ± 18.8	58.4 ± 13.8	< 0.001*
CTR (%) (TIC+ / TIC–)	48.0 ± 7.0	49.7 ± 6.5	0.070
Incidence of TIC			
Sex (Male / Female)	8.5% (29/342)	3.9% (19/482)	0.010*
Respiratory command (Old / New)	7.2% (39/545)	3.2% (9/279)	0.027*
Side of injection (Left / Right)	6.1% (14/228)	5.7% (34/596)	0.868
Contrast agent (≤ 350 / >350 mgI/mL)	3.9% (23/590)	10.7% (25/234)	< 0.001*
Severe TIC (CT_MPA_ < 150 HU)			
Age (years) (severe TIC+ / severe TIC–)	51.0 ± 12.4	66.3 ± 15.2	< 0.001*
Body weight (kg) (severe TIC+ / severe TIC–)	88.6 ± 26.8	58.9 ± 14.1	< 0.001*
CTR (%) (severe TIC+ / severe TIC–)	45.3 ± 5.6	49.7 ± 6.5	0.028*
Incidence of severe TIC			
Sex (Male / Female)	2.9% (10/342)	0.2% (1/482)	< 0.001*
Respiratory command (Old / New)	1.7% (9/545)	0.7% (2/279)	0.350
Side of injection (Left / Right)	1.3% (3/228)	1.3% (8/596)	1.000
Contrast agent (≤ 350 / >350 mgI/mL)	0.3% (2/590)	3.4% (8/234)	< 0.001*

Note. – Unless otherwise specified, means ± standards deviation are described for continuous variables. Comparisons were performed using the Fisher’s exact test and Student’s *t*-test for nominal and continuous variables, respectively. CT_MPA_ = CT attenuation of the main pulmonary artery; CTR = cardiothoracic ratio; HU = Hounsfield unit; TIC = transient interruption of contrast

* Statistically significant

Significant differences were observed in age (51.0/66.3 years for those with/without severe TIC [*p* < 0.001]), body weight (88.6/58.9 kg for those with/without severe TIC [*p* < 0.001]), and CTR (45.3%/49.7% for those with/without severe TIC [*p* = 0.028]) between patients with severe TIC and those without. Severe TIC incidence in male patients (2.9%) was significantly higher than in female patients (0.2%, *p* < 0.001). Severe TIC incidence in patients who received > 350 mgI/mL was also significantly higher than ≤ 350 mgI/mL (0.3%, *p* < 0.001). Severe TIC incidence in the new respiratory command (0.7%) tended to be lower than that in the old one (1.7%); however, the difference was not statistically significant (*p* = 0.350). There was no statistically significant difference in the incidence of severe TIC among scanners (1.2% [Canon-CT], 0.0% [Discovery 750 HD], and 2.1% [Revolution CT, *p* = 0.566]).

The CT_AORTA_/CT_PA_ was > 1.00 in 34.1% (186/545) and 28.3% (79/279) of patients for the old and new respiratory commands, respectively. CT_AORTA_ in patients who received contrast material in the right upper extremity (290.4 ± 77.3 HU) was significantly higher than that of patients who received from the left (271.3 ± 89.8 HU, *p* = 0.003). There was a significantly negative association between the CTR and CT_AORTA_ scores (*r* = -0.254, *p* < 0.001). There was a significantly positive association between CT_AORTA_ and CT_MPA_ (*r* = 0.203, *p* < 0.001).

### Combinations of significant factors on CT_MPA_ and TIC incidence analyzed using multivariable analysis

**Fig. 4:**
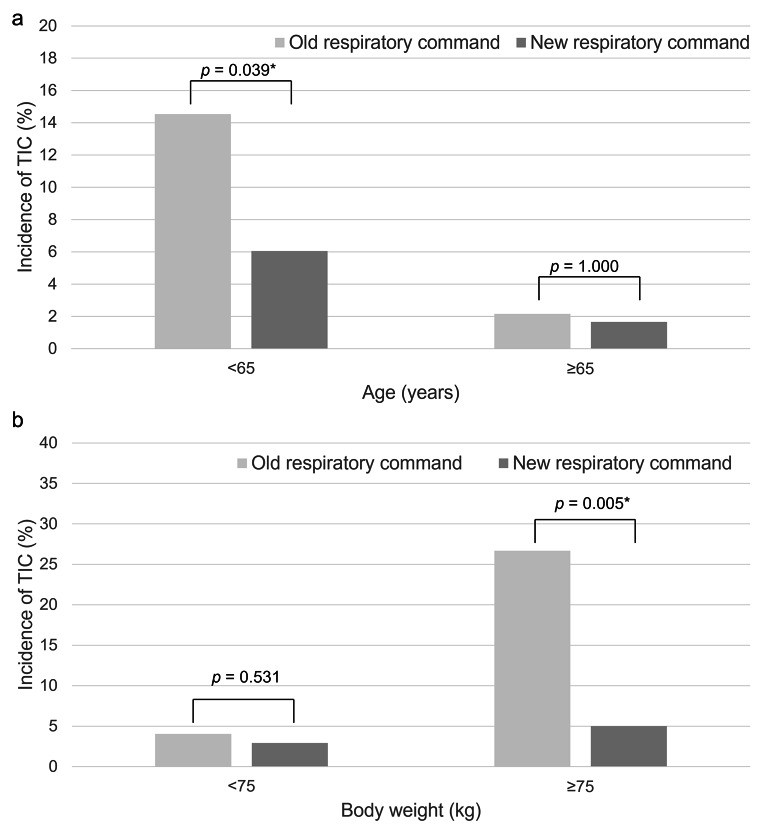
Impact of inspiratory command on the incidence of TIC in patient groups assorted by (**a**) age and (**b**) body weight. Comparison was performed with the Fisher’s exact test. * indicates statistically significant difference. TIC = transient interruption of contrast

Detailed results for the combinations of significant factors of CT_MPA_ and TIC incidence analyzed using multivariable analyses are provided in Table 5. Multiple regression analysis revealed that age, body weight, and respiratory command were significant factors associated with CT_MPA_. Logistic regression analysis revealed that the respiratory command, in addition to age and body weight was a significant factor in the incidence of TIC. By changing the respiratory command from end- to mid-inspiratory level, TIC incidence was significantly reduced in patients aged < 65 years (*p* = 0.039) and those with body weight ≥ 75 kg (*p* = 0.005) (Fig. 4). Regarding severe TIC incidence, only age, body weight, sex, and cardiothoracic ratio were significant factors.

**Table 5:** Combinations of parameters significantly associated with CT_MPA_ and incidence of TIC

	Parameters	Estimates	*p*-value
CT_MPA_	Age	1.520	< 0.001*
	Body weight	-2.296	< 0.001*
	Protocol (old/new = 0/1)	13.858	0.058
	(intercept)	378.111	
TIC (CT_MPA_ < 200 HU)	Age	-0.038	< 0.001*
	Body weight	0.050	< 0.001*
	Protocol (old/new = 0/1)	-0.943	0.022*
	(intercept)	-3.488	
Severe TIC (CT_MPA_ < 150 HU)	Age	-0.036	< 0.001*
	Body weight	0.049	< 0.001*
	Sex (male/female = 0/1)	-0.254	0.472
	Cardiothoracic ratio	-0.052	0.058
	(intercept)	-1.369	

Note. – For CT_MPA_ and TIC, the multiple regression analysis and logistic regression analysis, respectively were performed. CT_MPA_ = CT attenuation of the main pulmonary artery; HU = Hounsfield unit; TIC = transient interruption of contrast

* Statistically significant parameters

### Qualitative image analyses

There was no statistically significant difference in the median image quality score between the old and new respiratory command groups (*p* = 0.087). However, in patients with age < 65 years and those with body weight ≥ 75 kg, there were statistically significant improvements for the image quality score in the new respiratory command as compared with old respiratory command (*p* = 0.048 and 0.038, respectively) (Table 6). The median value for the interobserver agreement was 0.883.

**Table 6:** Percentages of patients for each image quality score

Patient group	Respiratory command	Score 4	Score 3	Score 2	Score 1	*p*-value
All	Old	78.9%	13.9%	5.7%	1.5%	0.087
	New	83.2%	15.1%	0.7%	1.1%	
Age < 65	Old	69.5%	18.6%	10.0%	1.8%	0.048*
	New	78.8%	19.2%	0.0%	2.0%	
Body weight ≥ 75 kg	Old	62.7%	21.3%	13.3%	2.7%	0.038*
	New	80.0%	17.5%	0.0%	2.5%	

Note. – Comparisons between old and new respiratory commands were performed with the Mann-Whitney U test

* Statistically significant

## Discussion

In this study, we found that the respiratory command, along with patient age and body weight, was a significant factor influencing the incidence of transient interruption of contrast. The incidence of transient interruption of contrast was significantly reduced with mid-inspiratory commands compared with end-inspiratory commands in patients aged < 65 years and those with body weight ≥ 75 kg who were at high risk for TIC.

TIC definition varied slightly across studies. Sudarski et al. used the aorto-pulmonary CT attenuation ratio as an indicator, which corresponds to CT_AORTA_/CT_MPA_ in our study, and patients with a value > 1.00 was regarded as having TIC. In their report, TIC incidence in the new respiratory command group was 21.6%, which was slightly lower than that reported in our study (28.3%). The definition of CT_AORTA_/CT_MPA_ > 1.00 stick to fundamentals of TIC. However, aorto-pulmonary CT attenuation ratio uses the CT attenuation of the aorta, which is affected by several factors. More specifically, the timing of the contrast material reaching the aorta is delayed in patients with lower cardiac function or those injected with contrast material from the left upper extremity. Furthermore, patients with sufficiently high CT_MPA_ values were regarded as having TIC when CT_MPA_ < CT_AORTA_. Owing to these problems, we used the CT_MPA_, which we believe is a clinically more relevant indicator, for the main analyses. When CT_MPA_ < 200 HU was defined as TIC, the TIC incidence at the end-inspiratory level was reported as 6.5–9.7% [11, 13], which is comparable with our results (7.2%).

It has been reported that younger patients are more likely to develop TIC [10], a phenomenon also observed in this study. Younger patients were significantly associated with a lower CT_MPA_ and a higher incidence of both TIC and severe TIC. Additionally, heavier patients were significantly associated with a lower CT_MPA_ and a higher incidence of TIC and severe TIC. Those results were also evidenced by the qualitative image analysis. In these patients, the amount of reflux blood volume might be relatively higher compared with injected contrast material volume, lowering the concentration of contrast material in the right side of the heart. Because the iodine density of the contrast agent was determined based on patient’s body weight, this factor was excluded according to the stepwise regression selection in the multiple regression analysis and logistic regression analysis.

Studies have investigated the effect of respiratory commands on TIC incidence [9–11]. However, these results are contradictory. Kay et al. reported that suboptimal opacification of the pulmonary artery was successfully reduced from 6.5 to 1.9% (*p* = 0.017). In another study of 231 patients, respiratory coaching did not have a significant effect (*p* = 0.61) [9]. A study with 225 patients reported that TICs were commonly observed even when patients were told to inspire gently during the scan [10]. In our study, which included 842 patients, change of respiratory command from end- to mid-inspiratory levels was found to significantly increase the CT_MPA_ and reduce TIC incidence. Our study is unique in that combinations of significant factors affecting CT_MPA_ and TIC were assessed using multivariable analyses. Changes in respiratory commands, patient age, and body weight were significant factors associated with CT_MPA_ and TIC incidence. In patients aged < 65 years and those with body weight ≥ 75 kg who were at high risk for TIC, the mid-inspiratory respiration command significantly reduced TIC incidence.

This study had some limitations. First, owing to its retrospective nature, patient alertness during the scan could not be assessed. Not all patients successfully followed the inspiratory commands; however, our data would reflect the incidence in actual daily clinical practice. Second, there were mixed CT scanning algorithms (dual-energy CT and conventional CT). However, a 70 keV monochromatic image, which corresponds to a 120 kVp image [12], was reconstructed, and the effect of this variation was minimal. On the other hand, our results would not be directly applicable to CT images scanned with lower tube voltages. Third, because a tube voltage of 100 kVp affects CT attenuation and was mostly used after changing the respiratory command, patients scanned at 100 kVp were excluded from this study. This was performed in order to align the conditions between mid-inspiratory and end-inspiratory command groups. Future studies are needed to assess the relationship between respiratory commands and TIC on low-tube-voltage CT pulmonary angiography. Fourth, because an optimal reference standard was not available for patients with TIC, a pulmonary embolism detection test was not performed. Future studies assessing the impact of respiratory command on the diagnosis of pulmonary embolism and clinical outcome would be necessary. Fifth, we did not exclude patients with patent foramen ovale, cardiac failure, and pulmonary hypertension from this study, because patients are not necessarily diagnosed as those diseases before CT pulmonary angiography and because such information was not necessarily available for all the patients in our study. Because hemodynamics of contrast material is different from normal in those patients, our results would not necessarily be applicable to them. Sixth, we used fixed delay [14] of 20 s in CT pulmonary angiography. Though, the scan delay falls within the optimal temporal window reported by Lee, et al. [15], our results may not necessarily be applicable to institutions where bolus tracking or test bolus technique are used in CT pulmonary angiography. Seventh, one radiologist read the images. However, intraclass coefficient among measurements obtained by two readers was almost perfect for CT_MPA_ and CT_AORTA_ values and interobserver agreement in qualitative image analysis was also high. Finally, our results are not applicable to patients who receive contrast material from the lower extremities, in whom image quality is degraded [16]. Further studies on these patients are required.

In conclusion, the respiratory command, as well as the patient age and body weight, had a significant impact on reducing TIC on CT pulmonary angiography. Mid-inspiratory respiration commands were also found to reduce TIC incidence in high-risk patients aged < 65 years and those with body weight ≥ 75 kg.
